# Rab11 modulates α-synuclein-mediated defects in synaptic transmission and behaviour

**DOI:** 10.1093/hmg/ddu521

**Published:** 2014-10-09

**Authors:** Carlo Breda, Marie L. Nugent, Jasper G. Estranero, Charalambos P. Kyriacou, Tiago F. Outeiro, Joern R. Steinert, Flaviano Giorgini

**Affiliations:** 1 Department of Genetics, University of Leicester, University Road, Leicester LE1 7RH, UK,; 2 MRC Toxicology Unit, University of Leicester, Lancaster Road, LeicesterLE1 9HN, UK,; 3 Department of NeuroDegeneration and Restorative Research, Center for Nanoscale Microscopy and Molecular Physiology of the Brain, University Medical Center Goettingen, Göttingen, Germany and; 4 Instituto de Medicina Molecular, Faculdade de Medicina da Universidade de Lisboa, Lisboa, Portugal

## Abstract

A central pathological hallmark of Parkinson's disease (PD) is the presence of proteinaceous depositions known as Lewy bodies, which consist largely of the protein α-synuclein (aSyn). Mutations, multiplications and polymorphisms in the gene encoding aSyn are associated with familial forms of PD and susceptibility to idiopathic PD. Alterations in aSyn impair neuronal vesicle formation/transport, and likely contribute to PD pathogenesis by neuronal dysfunction and degeneration. aSyn is functionally associated with several Rab family GTPases, which perform various roles in vesicle trafficking. Here, we explore the role of the endosomal recycling factor Rab11 in the pathogenesis of PD using *Drosophila* models of aSyn toxicity. We find that aSyn induces synaptic potentiation at the larval neuromuscular junction by increasing synaptic vesicle (SV) size, and that these alterations are reversed by Rab11 overexpression. Furthermore, Rab11 decreases aSyn aggregation and ameliorates several aSyn-dependent phenotypes in both larvae and adult fruit flies, including locomotor activity, degeneration of dopaminergic neurons and shortened lifespan. This work emphasizes the importance of Rab11 in the modulation of SV size and consequent enhancement of synaptic function. Our results suggest that targeting Rab11 activity could have a therapeutic value in PD.

## INTRODUCTION

Parkinson's disease (PD) is the second most common neurodegenerative disorder and affects ∼4% of the population over 80 years of age (1,2). Neuropathologically, this disorder is characterized by the presence of Lewy bodies (LBs) and Lewy neurites in dopaminergic neurons located in the *substantia nigra pars compacta*, and the loss of neurons in this region (3). The main protein component of LBs is α-synuclein (aSyn) (4). Point mutations and multiplications in the gene encoding aSyn lead to autosomal dominant versions of PD. In addition, genome-wide association studies suggest that polymorphisms in this gene increase susceptibility to sporadic forms of this disease (5). Although the precise cellular role of aSyn is still ambiguous, aSyn is located at the pre-synaptic termini (6) and has been implicated in modulating neurotransmitter release (7). Moreover, in pathological conditions, aSyn has been suggested to cause defects in several vesicle trafficking pathways (8).

Rab proteins are highly conserved small GTPases that orchestrate vesicle trafficking within a cell—with vesicle formation, movement, tethering and targeting controlled by specific Rabs (9). These proteins fluctuate between their target membranes and the cytosol dependent on their GTP-bound (active) or GDP-bound (inactive) status (10). Mutations in Rab genes have been associated with diverse neurological diseases (11). aSyn has been found to interact with several Rabs (12). Furthermore, aSyn disease-related pathology in model systems such as impaired endoplasmic reticulum (ER)–Golgi vesicle trafficking and loss of dopaminergic neurons is reversed by the overexpression of several Rabs, including Rab1, Rab3A and Rab8A (13–15). On the other hand, Rab11 has not been previously implicated in PD, although it has been linked to other neurodegenerative disorders (16–18). Rab11 is involved in the trafficking of vesicles between the recycling endosome and plasma membrane (19) and the *trans-*Golgi network (20). Rab11 also sequesters plasma membrane receptors and directs them to the recycling pathway, which plays a role in cell migration (21) and long-term potentiation (22).

The importance of Rab11 in the context of neurodegenerative disorders is emphasized by studies of Huntington's (HD) and Alzheimer's (AD) diseases. Indeed, Rab11 is functionally perturbed in several models of HD (23–26), and inhibition of Rab11 activity impairs vesicle formation from recycling endosomes in HD patient fibroblasts (24). Rab11 abrogates loss of dendritic spines in a primary neuronal model of HD, suggesting that Rab11 may play a critical early role in the synaptic dysfunction observed in HD (26). Rab11 overexpression also ameliorates synaptic dysfunction and neurodegeneration in a *Drosophila* model of HD (26,27). Regarding AD, direct interactions between Rab11 and the hydrophobic loops of presenilin 1 and 2 have been observed (28). Furthermore, oestrogen treatment has been found to divert Rab11 to the *trans*-Golgi network, thereby decreasing β-amyloid (Aβ) generation by promoting the budding of amyloid precursor protein-containing vesicles (29), while blockage of Aβ trafficking through Rab11 recycling vesicles enhances cellular Aβ accumulation (30).

Due to these many links between Rab11 and neurodegenerative disease, we hypothesized that Rab11 could play a role in modulation of PD pathogenesis. Here, we employ *Drosophila melanogaster* models of aSyn toxicity—and a panel of electrophysiological, immunohistochemical, genetic and behavioural analyses—to investigate the mechanistic role and therapeutic potential of Rab11 in PD. In a related recent study, we also demonstrated that Rab11 interacts with and modulates aSyn aggregation and secretion (31).

## RESULTS

### Rab11 normalizes aSyn-dependent potentiation of synaptic transmission at the *Drosophila* larval neuromuscular junction

**Figure 1.**
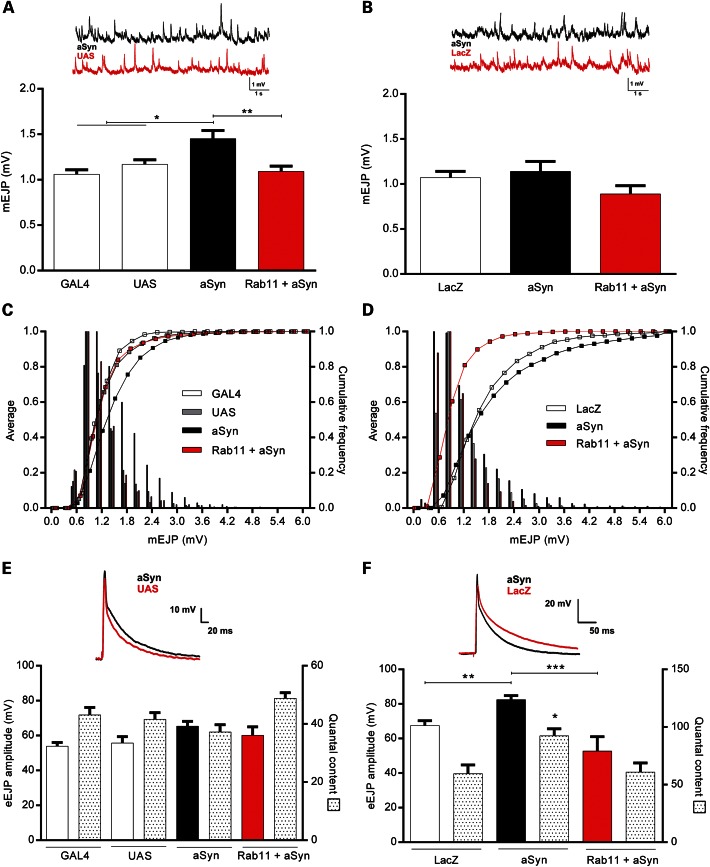
Rab11 reverses aSyn-dependent increases in average mEJP and eEJP amplitudes. Representative mEJP trace and summary graphs of averaged mEJP amplitudes for both Model 1 (**A**) and Model 2 (**B**) aSyn transgenic lines and their respective controls in third instar wandering larvae. Pan-neuronal expression of aSyn via the *elavGAL4* driver in Model 1 induced a strong increase in mEJPs in aSyn animals. Co-expression of Rab11 with aSyn returned the amplitudes back to control values (*N* = 8–19). No change was observed in Model 2 (*elavGAL4* driver) animals (*N* = 8–13). Relative cumulative frequency histograms and cumulative frequency curves for the mEJP amplitudes for both Model 1 (**C**) and Model 2 (**D**) aSyn transgenic lines and their respective controls are shown. eEJP sample recordings, summary graphs of averaged eEJP amplitudes and QC for Model 1 (**E**) and Model 2 (**F**) aSyn transgenic lines corrected for non-linear summation, which takes into account any changes in resting membrane potential. Pan-neuronal expression of aSyn via the *elavGAL4* driver in Model 2 aSyn animals induced an increase in eEJPs and QC (F; *N* = 6–13). Co-expression of Rab11 with aSyn led to a reduction and normalization of eEJP amplitudes in the φC31 animals. No change was observed with Model 1 larvae regarding eEJP amplitudes or QC (E; *N* = 5). Data are mean ± SEM. ANOVA with Newman–Keuls *post hoc* tests. *P < 0.05, **P < 0.01 and ***P < 0.001.

Expression of aSyn in flies yields several PD-relevant phenotypes, including formation of LBs, dopaminergic neuron loss and locomotor impairments (32). Here, we employed the GAL4/UAS system (33) to drive aSyn expression in specific tissues using two independent fly models carrying *UAS-aSyn* transgenes [Model 1 from (34) and Model 2 from (35); see Materials and Methods]. As we previously established that aSyn oligomers enhance basal synaptic transmission in rat hippocampal slices (36), we assessed whether the electrophysiological parameters of the neuromuscular junction (NMJ) in aSyn-expressing larvae mirrored these effects. Indeed, pan-neuronal expression of aSyn via the *elavGAL4* driver (*elav>aSyn*) in Model 1 strongly increased miniature excitatory junction potential (EJP) amplitudes (mEJP) from 1.17 ± 0.05 to 1.45 ± 0.09 mV (*P* < 0.05; Fig. 1A), with a similar pattern observed in Model 2, though this failed to reach statistical significance using ANOVA (Fig. 1B). More subtle effects on mEJP amplitudes in both models became apparent when analyzing mEJP distributions with the more sensitive Kolmogorov–Smirnov test (KS test; Fig. 1C and D; Model 1—UAS versus aSyn D = 0.2783, *P* < 0.0001; Model 2—LacZ versus aSyn D = 0.1478, *P* < 0.0001). Notably, co-expression of Rab11 with aSyn normalized these electrophysiological changes in both models and returned the mEJP amplitudes/distributions back to control values [(Fig. 1A; Model 1—*P* < 0.01, ANOVA) and (Fig. 1C and D; Model 1—aSyn versus Rab11 + aSyn D = 0.2729, *P* < 0.0001; Model 2—aSyn versus Rab11 + aSyn D = 0.2264, *P* < 0.0001, KS test)].

We also assessed evoked EJPs (eEJPs) with aSyn expression in these lines, and noted robust potentiation in Model 2 (*P* < 0.01; Fig. 1F). Model 1 larvae, on the other hand, exhibited no changes in eEJPs (Fig. 1E). We next analyzed the quantal content (QC) and found that QC was specifically increased in Model 2 flies, providing a rationale for the larger eEJPs observed (*P* < 0.05, Fig. 1F). Co-expression of Rab11 with aSyn led to a reduction and normalization of eEJP amplitudes and QC in these animals (*P* < 0.001 and <0.05, respectively; Fig. 1F), reiterating a modulatory role of Rab11 in aSyn-dependent potentiation of synaptic transmission.

### Rab11 ameliorates aSyn synaptic defects by restoration of synaptic vesicle size

**Figure 2.**
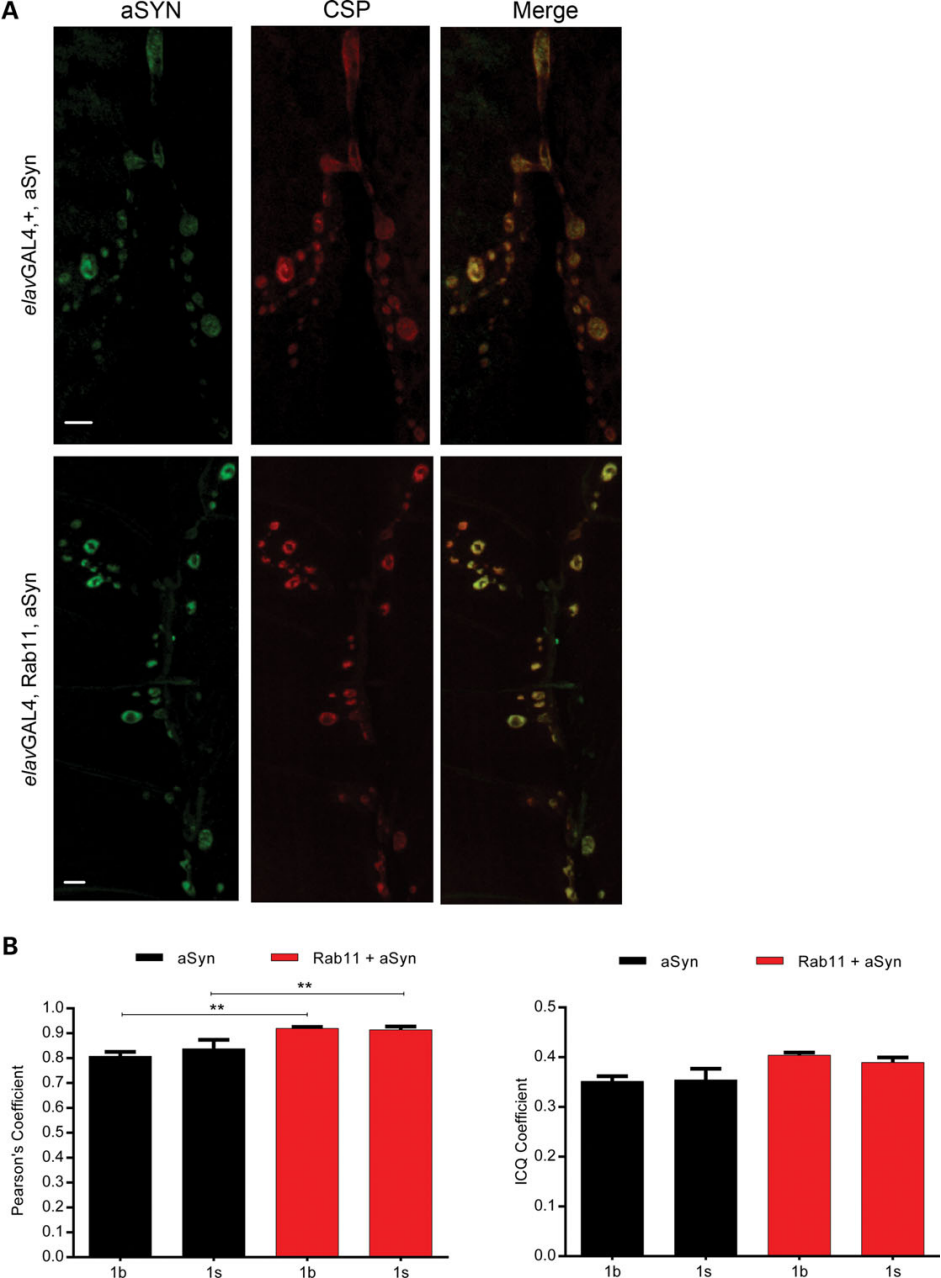
aSyn localizes to CSP in larval NMJ boutons. (**A**) Representative third instar larval NMJs of aSyn and Rab11 + aSyn individuals (*elavGAL4* driver with Model 1 line) immunostained with aSyn and CSP antibodies. Scale bar = 8 µm. (**B**) The spatial distribution of aSyn and CSP overlaps as indicated by Pearson's and ICQ coefficients. Mean ± SEM. ANOVA with Newman–Keuls *post hoc* tests. **P < 0.01.

To investigate the mechanism(s) underlying Rab11 modulation of aSyn-induced electrophysiological abnormalities at the NMJ, we next explored localization of Rab11 and aSyn in larval NMJs using immunocytochemistry. We found endogenous Rab11 to be highly expressed in segmental axonal termini, synaptic boutons and muscle tissues (Supplementary Material, Fig. S1A). Model 1 flies, *elav>aSyn*, exhibited robust aSyn levels at the NMJ (Fig. 2A). Significant co-localization between aSyn and cysteine-string protein (CSP)—an endogenous marker of synaptic vesicles (SVs) (37)—was observed in both bouton types (1s and 1b) found at the *Drosophila* NMJ [Fig. 2B, Pearson's coefficient = 0.852, intensity correlation quotient (ICQ) = 0.353]. Furthermore, using immunocytochemistry, we found aSyn enrichment in *elav>aSyn* expressing NMJs co-stained with the active zone containing scaffolding protein, bruchpilot (BRP) (38) (Supplementary Material, Fig. S1B). These data suggest that aSyn is associated with SVs and is localized to the synaptic active zones. In larvae overexpressing Rab11 we observed similar localization patterns for these proteins, though aSyn distribution appears to be shifted towards the membrane, and away from the centre of the bouton (Fig. 2 and Supplementary Material, Fig. S1B). A modest increase in overlap between aSyn and CSP was also observed (Fig. 2B, Person's coefficient = 0.916, ICQ = 0.396), indicating more aSyn is localized to SVs. No difference in aSyn*/*BRP co-localization was observed (Supplementary Material, Fig. S1B).

**Figure 3.**
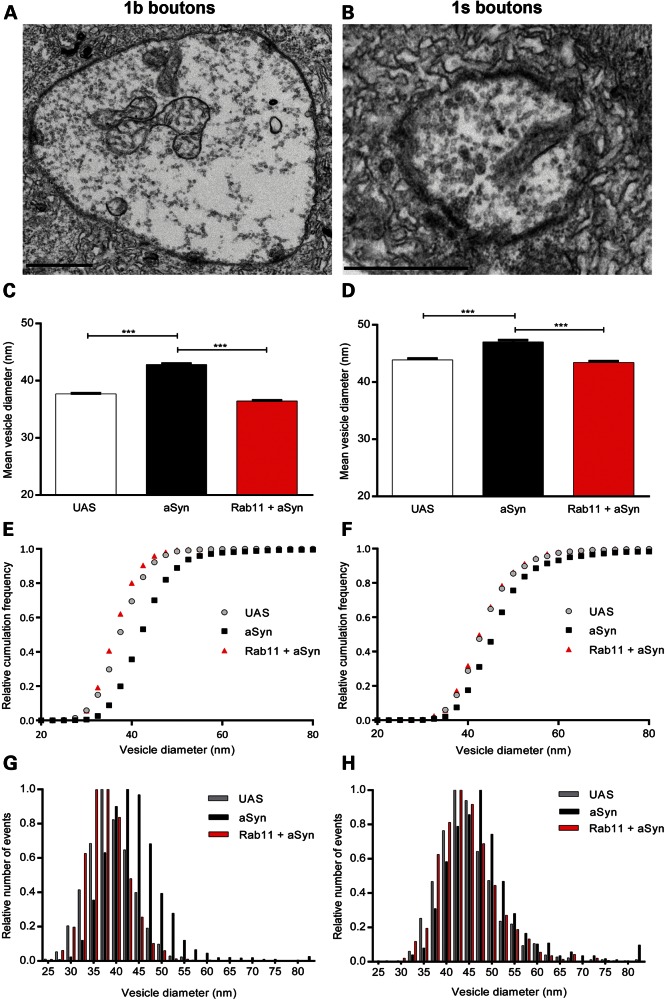
Increased SV size in aSyn larvae is rescued by Rab11 overexpression. Representative images of 1b (**A**) and 1s (**B**) boutons from WT NMJs. Average vesicle diameter in 1b (**C**) and 1s (**D**) boutons for UAS control, aSyn and Rab11 + aSyn larvae (Model 1 line). Increased vesicle size was observed upon expression of aSyn, which is fully reversed to control levels by Rab11 overexpression. Relative cumulative frequency plots and histograms and of 1b (**E** and **G**) and 1s (**F** and **H**) boutons; a right shift is observed in aSyn larvae in comparison with controls. SV size is rescued by overexpression of Rab11 in both boutons (1b boutons, *N* = 2737–3190 and 1s boutons, *N* = 804–1019). Data are mean ± SEM. ANOVA with Newman–Keuls *post hoc* tests. ***P < 0.001.

As these data suggest that localization of aSyn/Rab11 to SVs may play an important role in modulating the observed electrophysiological phenotypes, we next employed electron microscopy to determine SV size at the larval NMJ (Fig. 3A and B). Of the two bouton types found at the NMJ in fruit flies, 1s boutons display larger SVs, contain fewer mitochondria and active zones, and exhibit less enveloping subsynaptic reticulum (39). Notably, 1s boutons are proposed to play the major role in generation of synaptic activity in response to stimuli at the larval NMJ (39). SV diameters of both bouton types were determined in larvae expressing aSyn pan-neuronally, as well as in transgene controls. We observed a significant increase in SV diameters in 1b and 1s boutons in both transgenic lines expressing aSyn compared with the UAS controls (*P* < 0.001; Fig. 3C–H and Supplementary Material, Fig. S2), leading to a right shift in cumulative probability plots for SV size distribution (Fig. 3E and F, and Supplementary Material, Fig. S2C and D). The increased quantal responses at the larval NMJ due to aSyn could thus be explained by increased SV size. Notably, overexpression of Rab11 restored vesicle size in aSyn larvae (*P* < 0.001; Fig. 3C–H and Supplementary Material, Fig. S2), indicating that increased levels of Rab11 normalize SV size and thereby ameliorate the altered synaptic transmission due to aSyn.

### Rab11 ameliorates aSyn-dependent larval crawling impairments

**Figure 4.**
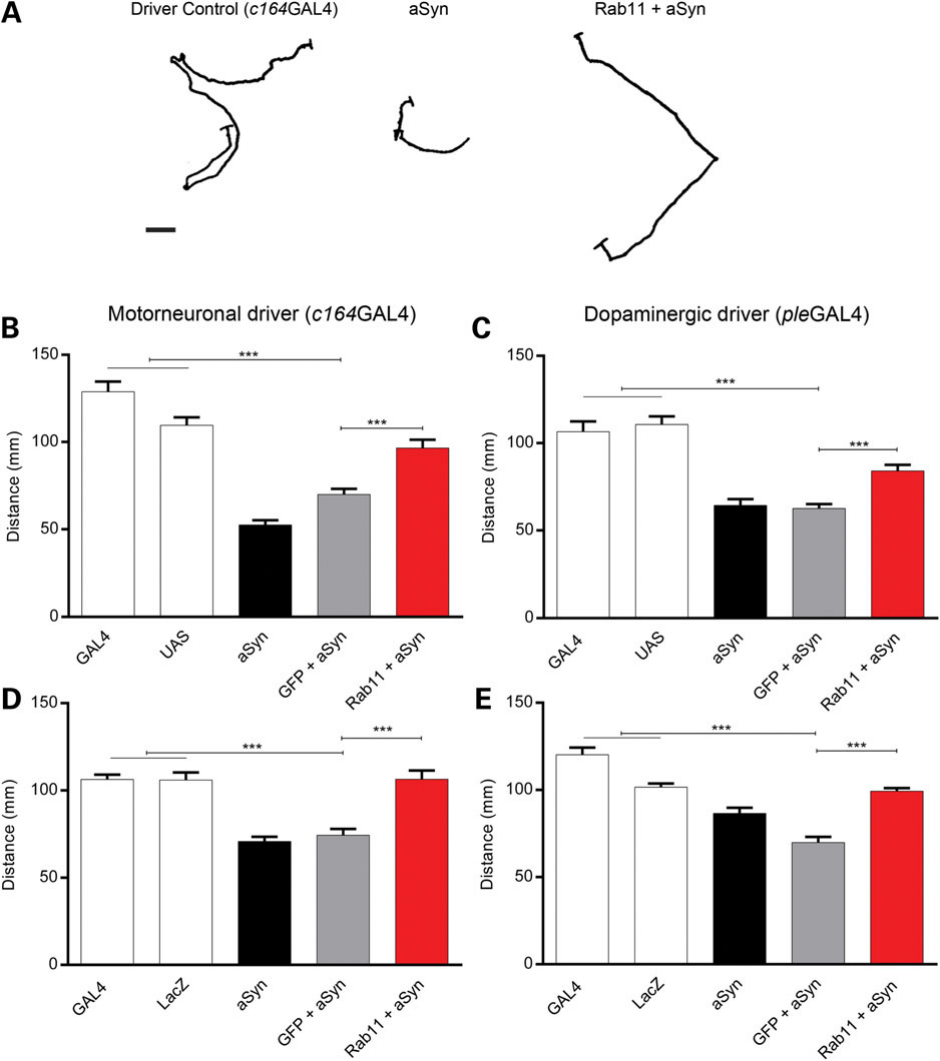
Rab11 overexpression rescues impaired locomotor behaviour in third instar larvae expressing aSyn. Representative crawling behaviour of control and larvae expressing aSyn, and Rab11 + aSyn in the motorneurons (**A**). Mean distance travelled by Model 1 larvae expressing aSyn, GFP + aSyn and Rab11 + aSyn in the motorneurons (**B**) and in the dopaminergic neurons (**C**). Similar crawling behaviour was observed in Model 2 larvae expressing aSyn in motorneurons (**D**) or dopaminergic neurons (**E**). Data are mean ± SEM. ANOVA with Newman–Keuls *post hoc* tests. ***P < 0.001. *N* = 30.

Third instar larvae display characteristic crawling behaviour comprising forward movements in a specific direction followed by pauses during which the surrounding area is sensed (40). We have previously observed defects in this crawling behaviour due to aSyn expression (15), and thus here employed this metric to monitor if the Rab11-dependent improvements in synaptic transmission at the NMJ led to consequent amelioration of locomotor impairments in larvae. Expression of aSyn in motor neurons using the *c164GAL4* driver (41) resulted in reduced distance travelled during a 2-min period relative to controls (*P* < 0.001; Fig. 4A, B and D). Strikingly, when Rab11 was co-expressed in aSyn larvae, crawling distance returned to control levels (*P* < 0.001; Fig. 4A, B and D). Larvae expressing aSyn in dopaminergic neurons (using the *pleGAL4* driver) also exhibited reduced crawling distance (*P* < 0.001; Fig. 4C and E), which was reversed by Rab11 co-expression (*P* < 0.001; Fig. 4C and E).

To further explore whether active Rab11 is required for the protection observed above, we also investigated the effect of the GDP-bound, dominant-negative Rab11 mutant (Rab11 S25N) (42) on the crawling behaviour of aSyn animals. We found that Rab11S25N expression did not rescue aSyn-dependent crawling defects (*P* = ns, Supplementary Material, Fig. S3A), and indeed, in wild type (WT), larvae caused a significant reduction in the distance crawled (*P* < 0.001; Supplementary Material, Fig. S3A). These data support the notion that active Rab11 is required for protection in aSyn flies.

### Rab11 reverses the loss of dopaminergic neurons in adult *Drosophila* brains

**Figure 5.**
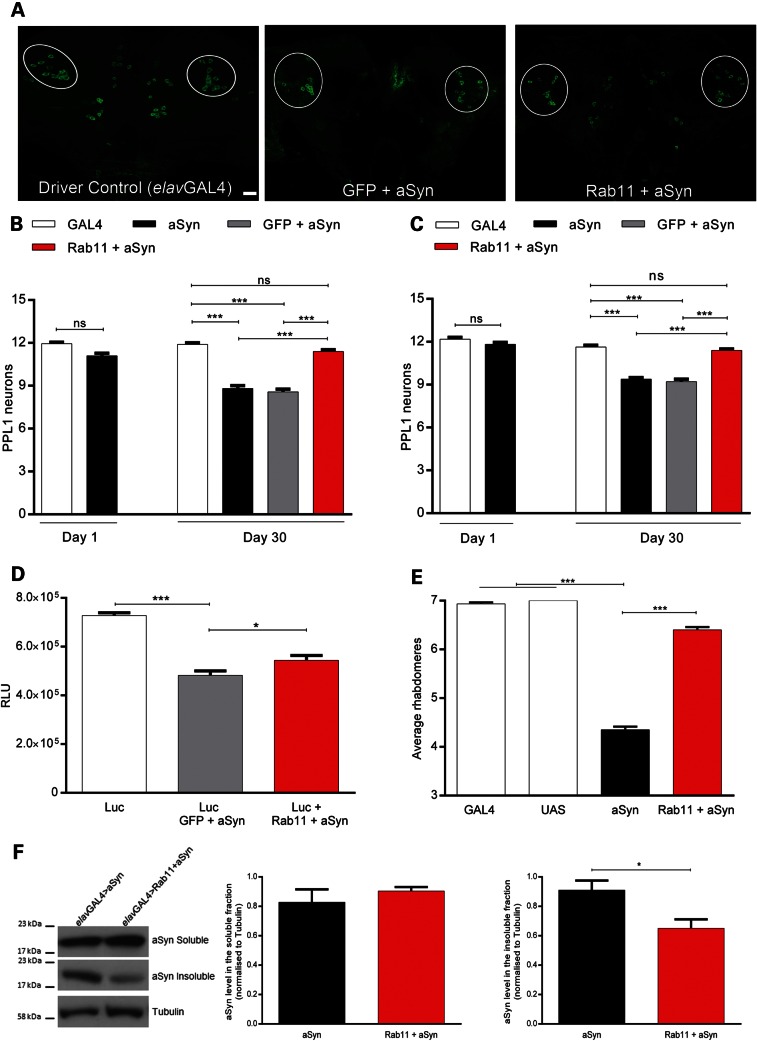
Dopaminergic neuron loss is reversed by Rab11 overexpression. Representative adult (Day 30) *Drosophila* posterior inferiorlateral protocerebrum confocal images (PPL1, circled) of control (left panel) and Model 1 flies expressing GFP + aSyn (middle panel) or Rab11 + aSyn (right panel), driven by *elavGAL4* and immunostained with anti-TH antibody. Scale bar = 40 µm. (**A**) Numbers of PPL1 neurons in flies at Day 1 and Day 30 (*elavGAL4* in **B** and *pleGAL4* in **C**). The expression of aSyn and GFP + aSyn by both drivers decreases the number of PPL1 cells, which is restored to normal levels by the overexpression of Rab11 (*N* = 19–65 hemispheres). Luciferase intensity measurement in Model 1 flies expressing aSyn in dopaminergic neurons (**D**). The reduction in luciferase signal is restored by the overexpression of Rab11 (*N* = 4). Quantification of average rhabdomeres *per* ommatidum in aSyn flies with and without photoreceptor Rab11 overexpression at Day 30 (**E**). Expression of aSyn via *gmrGAL4* leads to a reduction in the number of photoreceptors in Model 1 flies, whereas Rab11 overexpression prevents this neurodegeneration (*N* = 60–136 ommatidia). (**F**) aSyn levels in *elav*-driven aSyn and Rab11 + aSyn fly heads. Representative immunoblot of aSyn soluble and insoluble fractions (left panel). The graphs represent immunoblot quantification of aSyn in the soluble (middle panel) and insoluble (right panel) fractions. The overexpression of Rab11 reduces the level of aSyn in the insoluble fraction, whereas no changes are found in the soluble fraction (*N* = 4). Data are mean ± SEM. ANOVA with Newman–Keuls *post hoc* tests. *P < 0.05, ***P < 0.001 and ns = not significant.

Having found that Rab11 ameliorates PD-relevant phenotypes during development, we next sought to extend these findings into adult flies. Previous studies have shown that expression of aSyn causes the degeneration of tyrosine hydroxylase (TH)-expressing dopaminergic neurons in aged adult flies (34,43). We thus analyzed the protocerebral posterior inferiorlateral (PPL1) subset of TH neurons located in the adult *Drosophila* posterior brain, which is particularly sensitive to aSyn expression (Fig. 5A) (15). We found that pan-neuronal expression of aSyn led to loss of these neurons (*P* < 0.001; Fig. 5B and Supplementary Material, Fig. S4A), which was reversed by overexpression of Rab11 (*P* < 0.001; Fig. 5B and Supplementary Material, Fig. S4A). Similarly, a progressive loss of dopaminergic neurons was observed when aSyn expression was limited to TH-positive neurons using the *pleGAL4* driver, with 30-day-old flies displaying marked neuronal loss (*P* < 0.001; Fig. 5C), which again was ameliorated upon Rab11 co-expression (*P* < 0.001; Fig. 5C).

To support our immunocytochemical data, we employed a luminescence-based assay in which luciferase was co-expressed with aSyn as a further readout for neuronal dysfunction/death in these flies. A reduction in luciferase signal was found in flies expressing aSyn both pan-neuronally (*P* < 0.05, Supplementary Material, Fig. S4B) and specifically in the TH-positive neurons (*P* < 0.001, Fig. 5D). The overexpression of Rab11 in both conditions increased the luciferase signal (*P* < 0.05 for both drivers; Fig. 5D and Supplementary Material, Fig. S4B), indicating neuronal rescue. Taken together, these results suggest that increased Rab11 expression protects dopaminergic neurons in the *Drosophila* central nervous system from aSyn toxicity.

We next assessed whether Rab11 protects against aSyn-dependent neurodegeneration more generally. For these experiments, we drove expression of aSyn with the eye-specific *gmrGAL4* driver and evaluated degeneration of photoreceptor neurons using the pseudopupil assay, a widely used readout in several fly neurodegeneration models (34,44,45). We found that aSyn expression led to a ∼62% reduction in rhabdomeres in 30-day-old flies (*P* < 0.001, Fig. 5E), and that co-expression of Rab11 restored rhabdomere number to approximately WT levels (*P* < 0.001 versus aSyn; Fig. 5E). Thus, Rab11 is able to generally reverse aSyn-dependent neurodegeneration, regardless of neuronal population targeted.

Finally, in order to elucidate potential protective mechanisms underlying Rab11 protection, we assessed the level of soluble/insoluble aSyn in adult heads at Day 30 post-eclosion as a measure of aSyn aggregation. Importantly, we found that pan-neuronal expression of Rab11 significantly decreased levels of insoluble aSyn (*P* < 0.05, Fig. 5F).

### Rab11 ameliorates disease-relevant phenotypes in adult flies expressing aSyn

**Figure 6.**
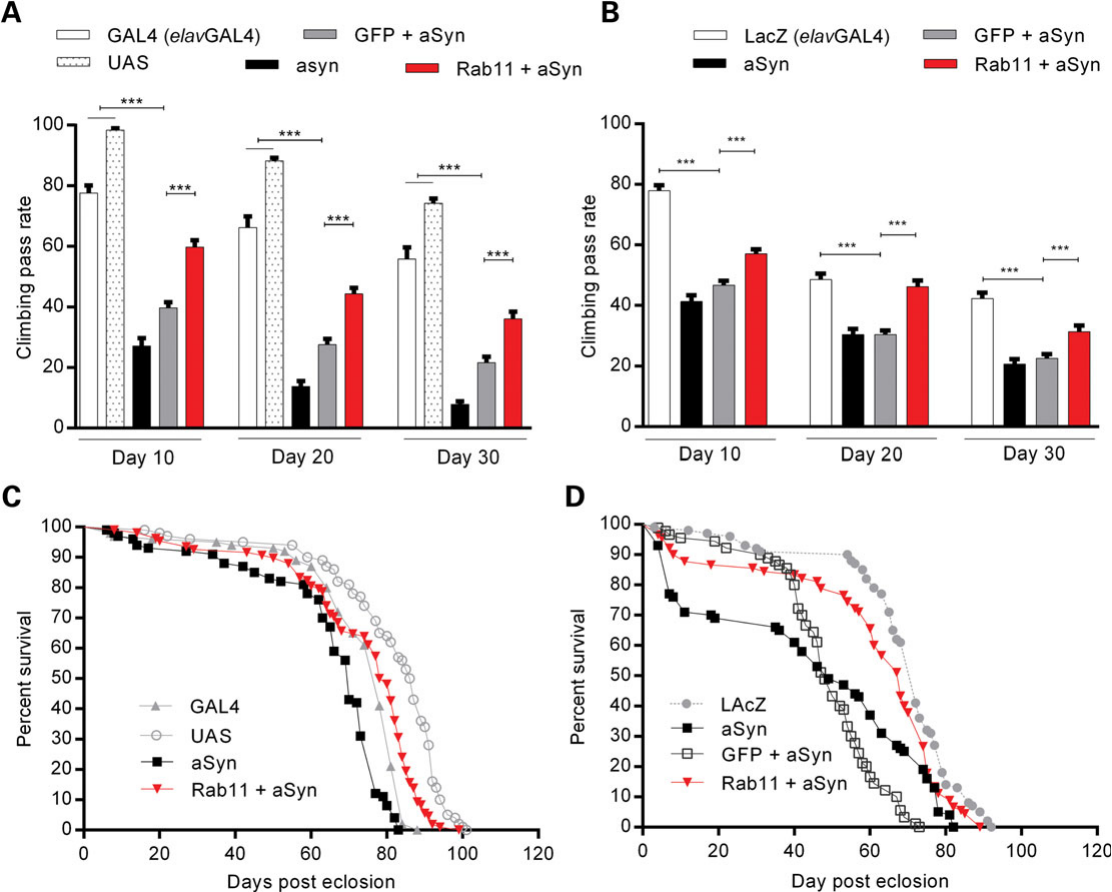
aSyn-dependent adult phenotypes are ameliorated by Rab11 overexpression. Mean climbing pass rate for Model 1 (**A**) and Model 2 (**B**) flies expressing aSyn, GFP + aSyn and Rab11 + aSyn pan-neuronally by the *elavGAL4* (*N* = 50–60 per condition). aSyn expression causes a reduction in climbing at all post-eclosion ages, which are strongly rescued by Rab11 overexpression. Lifespan was evaluated in Model 1 and Model 2 flies expressing aSyn pan-neuronally (**C** and **D**, respectively, *N* = 100 per genotype). aSyn expression increases mortality, which is reversed by Rab11 overexpression (*P* < 0.001 for both models). Data are mean ± SEM. ANOVA with Newman–Keuls *post hoc* tests. **P < 0.01, ***P < 0.001 and ns = not significant.

We next analyzed locomotor impairments due to pan-neuronal and dopaminergic expression of aSyn by assessing negative geotaxis (climbing) in aged flies (Fig. 6A and B, and Supplementary Material, Fig. S5), as locomotor activity is linked to dopaminergic neurons (46). We found that pan-neuronal expression of aSyn led to a significant decrease in climbing relative to controls at all post-eclosion ages tested (*P* < 0.001; Fig. 6A and B). The overexpression of Rab11 rescued this impairment at all ages (*P* < 0.001). A similar deficiency in climbing was displayed by flies in which aSyn was selectively driven in dopaminergic neurons, with a decrease in climbing observed at Day 20 and 30 (*P* < 0.001, Supplementary Material, Fig. S5), and an improvement in behaviour observed upon overexpression of Rab11. We also evaluated the climbing phenotypes of Rab11S25N + aSyn flies, and found that overexpression of Rab11 S25N significantly decreased climbing of aSyn flies at all ages tested (*P* < 0.001; Supplementary Material, Fig. S6).

Finally, we assessed if Rab11 overexpression could ameliorate the shortened lifespan exhibited by aSyn flies. We found that pan-neuronal Rab11 expression reversed the reduced survival observed in both aSyn models (*P* < 0.001; Fig. 6C and D), extending median lifespan from 70 to 79 days (Fig. 6C), and from 48 to 68 days, respectively (Fig. 6D).

## DISCUSSION

Although the exact function of aSyn is not fully understood, some studies suggest that it is linked to synaptic transmission. This is emphasized by its localization to pre-synaptic termini (47) and its involvement in several steps of the neurotransmission machinery. The overexpression of aSyn in murine dopaminergic neurons leads to inhibition of TH activity with a consequent reduction in dopamine synthesis (48,49). Moreover, aSyn association with the distal reserve pool of SVs and the SNARE complex indicate a physiological role of aSyn in the recycling of SVs and their fusion with the plasma membrane (6,50). aSyn has also been found to interact with several Rab GTPase proteins, which are highly conserved regulators of intracellular membrane trafficking between organelles (9). aSyn associates with Rab3a, Rab5 and Rab8 in dementia with LB patients and mouse models (12,51), and an interaction between endocytosed aSyn and Rab11a has been observed in mammalian cells (52). Notably, Rab1 and Rab8a overexpression reverses aSyn-dependent impairment of ER–Golgi transport in yeast, fruit flies and *Caenorhabditis elegans* (13–15).

Synaptic dysfunction occurs in several human neurodegenerative diseases prior to neuronal loss and the manifestation of deficient behaviours (53). For this reason, we first investigated the effect of aSyn toxicity in *Drosophila* larvae. We found that synaptic transmission was altered at the NMJ, and that this correlated with an enlargement of SVs. These physiological effects had a negative consequence on larval crawling behaviour when aSyn was expressed either in the motorneurons or in the dopaminergic neurons. Locomotor dysfunction was maintained through to adults, as evidenced by decreased climbing at all post-eclosion ages tested. Finally, the survival of flies expressing pan-neuronal aSyn was diminished compared with the controls. Strikingly, for all these aSyn-mediated abnormalities, overexpression of *Drosophila* Rab11 significantly ameliorated these mutant phenotypes.

We recently demonstrated that Rab11 similarly rectifies several phenotypes in a fruit fly model of HD, including compromised spontaneous miniature and evoked transmission and SV size (27), although these effects were in the inverse direction to those observed in the current study with aSyn flies. The ability of Rab11 to normalize SV size in both experimental paradigms indicates that it plays a critical role in homeostasis of SV size. Indeed, several Rab (-interacting) proteins have been suggested to act as mediators of synaptic homeostasis (54,55). Supporting our observations here, aSyn overexpression in primary mouse neurons causes an enlargement in SVs (56). We further found that the increased amplitude of miniature events correlated with larger SV diameters.

**Figure 7.**
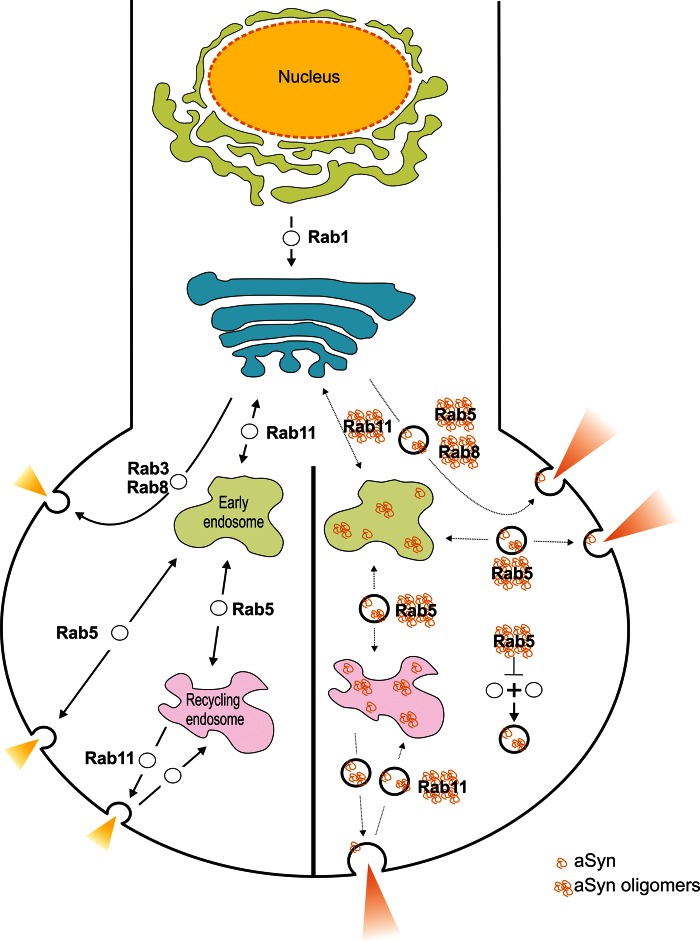
Model suggesting how aSyn may alter normal synaptic function of Rab GTPases. Left panel: normal synaptic transmission. Right panel: aSyn expression causes an enlargement of SVs by sequestering Rab5, which is normally involved in preventing homotypic fusion between SVs. Alternatively, aSyn may function as a scaffold protein attracting and promoting the fusion between multiple vesicles. Either mechanism could lead to an increase in synaptic transmission. aSyn interaction with Rab11 reduces its activity and the vesicle trafficking associated with it. The overexpression of Rab11 reverses these defects, by reducing aSyn aggregation and enhancing its secretion, therefore restoring normal vesicle trafficking.

How does aSyn expression lead to SV enlargement? A possible explanation can be found in the propensity of aSyn to interact with Rab5 (57). Rab5 plays a key role preventing the fusion of SVs with each other—known as homotypic fusions (57). Therefore, by sequestering or negatively interacting with Rab5, aSyn may lead to increased fusion between SVs resulting in their enlargement (Fig. 7). Alternatively, aSyn may function as a scaffold protein attracting and promoting the fusion between multiple vesicles. This is in agreement with the observation that the expression of aSyn in yeast causes vesicle accumulation (58). aSyn has also been shown to interact with clathrin signalling (59,60) and clathrin together with its adaptor proteins is involved in regulating vesicular size. This could point towards a possible regulation of synaptic transmission via aSyn. The possibility that the aSyn-dependent increases of SVs, miniature events and evoked release occur by promoting the assembly of the SNARE complex, with its role in fusion mechanisms with the plasma membrane (50), should also be considered.

Interestingly, we observed differences in eEJPs between aSyn models—while Model 2 aSyn larvae exhibited potentiation of these amplitudes, Model 1 larvae displayed no change. This could be a consequence of homeostatic and compensatory down-regulation of evoked transmission in response to the greater miniature events observed in the Model 1 aSyn animals. Similar mechanisms of homeostasis have been shown as an upregulation of QC following genetic or pharmacological reduction in postsynaptic quantal responses (61,62).

It is clear from our work that aSyn perturbs—and that simultaneous Rab11 overexpression restores—vesicle trafficking. In this regard, Rab3—a GTPase localized to SVs—has been implicated in synaptic homeostasis (54). Given the ubiquitous expression of Rab11 and its localization to synaptic boutons (63), it is possible that Rab11 performs a similar function at the synapse. In this scenario, aSyn would perturb the fine balance in synaptic transmission by interfering directly with Rab11 or with some effectors that regulate its function (Fig. 7). The overexpression of Rab11 would thereby compensate this dysfunction, shifting the system to a more ‘normal’ state. Recent work indicates that aSyn interacts with the GTP/GDP-binding pocket of Rab8a, abrogating its function (15). It is thus possible that aSyn may have a similar negative impact on the inactive–active state of Rab11. Enhanced Rab11 activity could also modulate the endosomal recycling rate in aSyn flies and thereby alter delivery of vesicle-required proteins to the membrane. Notably, Rab11 has been shown to interact with the ε subunit of the vacuolar type H^+^-ATPase (64), and a possible enhanced interaction with the vesicular H^+^-ATPase could alter vesicular parameters and rescue aSyn-dependent defects.

aSyn present in vesicles has an increased propensity to aggregate compared with its cytosolic counterpart (65). Thus, aSyn inclusion formation due to accumulation of defective vesicles may serve as an initial signal for enhancing further aggregation processes. This aSyn cluster may then sequester several proteins—including Rab11—depleting cellular factors involved in the diverse physiological processes. Interaction between aSyn and Rab11 has been demonstrated in parallel work conducted in a human cell model of PD by co-immunoprecipitation and co-localization studies (31). Importantly, Rab11 expression was found to decrease the number of aSyn inclusions and cellular toxicity, likely due to enhanced aSyn secretion from the cells (Fig. 7), which may explain the alterations in aSyn localization observed at the larval NMJ due to Rab11 overexpression. Our results from aged *Drosophila* heads support this role for Rab11 in the clearance of toxic insoluble aSyn, and further show that its protective properties depends on its activity, as the Rab11 dominant-negative variant failed to rescue any of the phenotypes assessed. Although the mechanisms are not fully understood, aSyn can actively be secreted from neurons via the endocytic pathway, exosomes and the ER/Golgi-to-plasma membrane secretory pathway (65,66).

This study shows for the first time that Rab11 can modulate aSyn-dependent synaptic dysfunction and neurodegeneration. We have found that Rab11 robustly ameliorates PD-related phenotypes induced by aSyn overexpression. Thus, small molecules which increase Rab11 activity may have therapeutic relevance for PD (67). Moreover, as Rab11 has been implicated in both Huntington's and Alzheimer's diseases, such potential drug strategies may have a general clinical value for neurodegenerative diseases.

## MATERIALS AND METHODS

### *Drosophila* genetics

Fruit flies were maintained on standard maize food at 25°C in a light/dark cycle of 12 : 12. The *elavGAL4* [c155], w; +; pleGAL4 (8848), w; UASRab11-GFP; + (8506), *w*; +; *UASaSyn* (8146), w; UASeGFP; + (5431) and *yw*; *UASRab11-YFP-S25N*; + (23261) fly stocks were obtained from the Bloomington Stock Center (Bloomington, Indiana). The *UASluc* line was a gift from Norbert Perrimon (Harvard Medical School). The c164GAL4 driver line was a gift from Juan Botas (Baylor College of Medicine). The *UASlacZ* and *UASaSyn* φC31 transgenic lines were kindly provided by Alf Herzig (Max-Planck-Institut für Biophysikalische Chemie). We employed two transgenic models for aSyn expression. The first model (Model 1) exploits a transgene encoding WT aSyn, which is inserted randomly into the genome using P-element-based transgenesis (34), whereas the second model (Model 2) harbours a transgene encoding WT aSyn targeted to the attP landing site in the 3R-86Fb genomic region using the φC31-based site-specific recombination system (35). Flies carrying *UASlacZ* targeted to the same location were used as controls.

### Behavioural assays

For larval crawling experiments, crosses were set up on standard maize fruit fly food mixed with 0.05% Bromophenol Blue (FisherBiotech) as described previously (27). Young, deep blue-coloured third instar wandering larvae were used for the crawling assay. Each larva was washed in distilled water and placed in the middle of a 145 mm petri dish where 0.8% agarose was previously poured and set. The distance covered by the larva in 2 min was manually tracked on a transparent paper placed on the top of the petri dish lid. The tracks were scanned and the distance calculated using ImageJ (68).

Negative geotaxis assays were performed as described (69). Briefly, flies carrying the desired genotype were placed in a cylinder consisting of two empty tubes taped together along the centre (with a diameter of 2.3 cm and a total length of 18.4 cm). An 8 cm line was drawn from one end identifying the threshold that flies must reach in order to pass. Before the experiment, flies were left to acclimatize for 1 min. For each trial, the tubes were tapped gently in order to send all the flies to the bottom. The flies were then allowed to fly or scale the sides of the tube for 10 s and the number of flies which passed the threshold recorded. The same cohort of flies was retested 10 times. In between climbing trials, the flies were allowed to rest for 1 min. This process was repeated on Day 10, 20 and 30 post-eclosion.

For longevity, females carrying the desired genotype were collected and kept in groups of 10 in separate vials. Vials were inspected and changed every 2–3 days, and the number of flies remaining alive was scored.

### Electrophysiology

Current clamp recordings using sharp electrodes [using pClamp 10, an Axoclamp 700B amplifier and Digidata 1321A (Molecular Devices, USA)] were made from ventral longitudinal muscle 6 in abdominal segments 2 and 3 of third instar wandering larvae in haemolymph-like (HL-3) solution containing 1.5 mm Ca^2+^ as described previously (70). Recording electrodes (10–20 MΩ) were filled with 3 M KCl. All eEJP/mEJPs were recorded from muscles with resting potentials more negative than −60 mV and at 22°C as differences in recording temperature cause changes in glutamate receptor kinetics and amplitudes (71). All eEJP amplitudes were corrected for non-linear summation (72). Larval rearing temperatures were 25°C to avoid temperature-dependent fluctuations of NMJ morphology and evoked responses (73). QC was calculated by dividing the mean eEJP amplitudes by the mean mEJP amplitude of a given cell. mEJPs and eEJPs were low-pass filtered at 1 kHz. Data were collected from averaged multiple eEJPs and 60 s of mEJP recordings per muscle.

### Determination of SV size

Preparation of NMJ tissues for electron microscope imaging was performed as previously described (27). About 1000 SV diameters were evaluated using the ImageJ software from four larvae for each genotype and analyzing at least six independent boutons per animal.

### Immunocytochemistry

For larval immunostaining, third instar larvae were dissected and immunostained as described (74). Briefly, larvae were collected and dissected in ice-cold 1× PBS before being fixed for 20 min in 4% formaldehyde–PBS. Samples were washed twice for 15 min in 0.1% PBST (1× PBS + 0.1% Triton-X), followed by 2× 30 min washes with 0.2% BSA in 0.1% PBST (PBTB) and 2 × 15 min washes with 2% goat serum in PBTB (PBTN). Samples were incubated overnight at 4°C with primary antibody. CSP (mouse, 1 : 100, Developmental Studies Hybridoma Bank), nc-82 (mouse, 1 : 100, Developmental Studies Hybridoma Bank), aSyn (rabbit, 1 : 100, Millipore) and HRP (rabbit, 1 : 100, Jackson ImmunoResearch) were used. Larvae were then washed 2 × 15 min with PBTB and incubated for 30 min with PBTN. Rabbit Cy2 and mouse Cy5 (1 : 200, Abcam) were used as secondary antibodies and incubated at room temperature for 2 h. Finally, samples were washed 2 × 15 min with PBTB and mounted in 3% *n*-propylgallate + 80% glycerol PBS solution.

For immunocytochemistry of adult brains, 15 virgin females were collected at a specific age and fixed overnight in 4% formaldehyde–PBS. Brains were dissected in ice-cold PBS and permeabilized via 3 × 20 minute washes with 1% PBST followed by a wash in a blocking solution made of 10% goat serum in 0.5% PBST for at least 1 h. Samples were incubated with mouse TH antibody (1 : 100, Immunostar) overnight at 4°C. Unbound antibody was removed by 3 × 20 min washes in 0.5% PBST before being incubated for 2 h at room temperature with secondary antibody (Anti-mouse Cy5, 1 : 500). After 3 × 20 min washes with 0.5% PBST, brains were mounted in 3% *n*-propylgallate + 80% glycerol PBS solution.

Rhabdomere immunostaining was performed by removing fly heads and fixing overnight at 4°C in 4% formaldehyde–PBS. Heads were washed 3 × 5 min in PBS prior to dissection. Eyes were removed from the brain and washed 3 × 20 min in 1% PBST (Triton-X). Samples were incubated for 30 min in 10% goat serum in 0.5% PBST for at least 1 h followed by a 4°C overnight incubation with Alexa Fluor^®^ 488 Phalloidin (1 : 200, Molecular Probes). Eyes were then washed 3 × 20 min with 0.5% PBST and mounted in 3% *n*-propylgallate + 80% glycerol PBS solution. All samples were visualized on an Olympus FV1000 confocal microscope and processed with an Olympus software.

### Luciferase assays

Aged flies were collected in quadruplicate groups of 10 virgin females and frozen in liquid nitrogen. Proteins were extracted following manufacturer's instructions (Luciferase Assay System, Promega). Briefly, flies were placed in 200 µl of cell lysis buffer and homogenized in a 1.5 ml microcentrifuge tube. Samples were subsequently subjected to three cycles of freezing in dry ice and thawing at 37°C, followed by centrifugation at 9300 *g* for 10 min and collection of lysis supernatants. Protein concentrations were determined by Bradford's Reagent (Sigma-Aldrich) and used for normalization. To quantify luciferase activity, 20 µl of each fly sample was distributed in a 96-well plate and 100 µl of Luciferase System reagent was injected in each. Luminescence was detected using a Fluorostar Omega microplate reader (BMG Labtech).

### Immunoblotting

Insoluble aSyn was prepared as described in ref. (75) with minor modifications. Hundred heads of 30-day-old flies were collected and homogenized using a sterile plastic pestle in TNE buffer (10 mm Tris–HCl, pH 7.4, 150 mm NaCl and 5 mm EDTA) containing protease inhibitors (Complete Mini; Roche) and detergents (0.5% Nonidet P-40). The homogenate was centrifuged (30 min at 100 000*g* at 4°C), and the resulting pellet (P1) and supernatant (S1, soluble) fractions were collected. The P1 was resuspended in the same TNE/NP-40 buffer followed by centrifugation at 100 000 *g* for 1 h at 4°C. The pellet was taken as the insoluble fraction and solubilized in TNE buffer containing 1% SDS. S1 and P2 samples were quantified using Bradford's Reagent (Sigma-Aldrich) and normalized. Proteins were loaded on SDS–PAGE gels, transferred onto nitrocellulose membranes (Whatman), blocked in 5% powdered milk and incubated with the primary antibody, either mouse anti-aSyn (1 : 1000, BD transduction Laboratories) or mouse anti-tubulin (1 : 10 000, Sigma). Horseradish peroxidase-conjugated goat anti-mouse (1 : 10 000; Vector Labs) was used as the secondary antibody and signals were visualized using SuperSignal^®^ West Dura Extended duration Substrate (Thermo Scientific).

### Statistical analysis

Statistical analysis was performed using Prism 6 (GraphPad Software). In general, analysis was carried out using ANOVA with the Newman–Keuls *a posteriori* test. mEJP amplitude distributions were analyzed using the KS test in Prism 6 (GraphPad Software). For longevity, survival curves were generated and data were analyzed using the Kaplan–Meier method and log-rank statistics in Prism 6 (GraphPad Software).

## AUTHORS' CONTRIBUTIONS

C.B., F.G., M.L.N. and J.R.S. designed the experiments. C.B. and J.G.E. performed the *Drosophila* experiments. M.L.N. performed the electrophysiological experiments. C.B., F.G., M.L.N., J.R.S. and C.P.K. analyzed the data. C.B., F.G. and J.R.S. wrote the manuscript. C.P.K. and T.F.O. edited the manuscript.

## SUPPLEMENTARY MATERIAL

Supplementary Material is available at *HMG* online.

## FUNDING

C.B. was supported by a grant from Parkinson's UK (G-1203) to F.G., T.F.O. and C.P.K. M.L.N. was supported by a PhD studentship funded by the Biotechnology and Biological Sciences Research Council (BBSRC). J.R.S. is supported by the Medical Research Council (MRC). T.F.O. is supported by the DFG Center for Nanoscale Microscopy and Molecular Physiology of the Brain, Germany. Funding to pay the Open Access publication charges for this article was provided by Parkinson's UK.

## Supplementary Material

Supplementary Data
