# Supplementary material for: Rab11 modulates α-synuclein-mediated defects in synaptic transmission and behaviour
Source: Hum Mol Genet. 2014 Oct 9;24(4):1077–91. doi: 10.1093/hmg/ddu521 (PMC4986550; doi:10.1093/hmg/ddu521)
Supplement: Supplementary Data [file supp_24_4_1077__index.html]

Rab11 modulates α-synuclein mediated defects in synaptic transmission and behaviour — Rab11 modulates α-synuclein-mediated defects in synaptic transmission and behaviour — Rab11 modulates α-synuclein-mediated defects in synaptic transmission and behaviour — Supplementary Data 

# Rab11 modulates α-synuclein-mediated defects in synaptic transmission and behaviour

## Supplementary Data

Supplementary Data

**Files in this Data Supplement:**

- Supplementary Data - Pdf file
