# Supplementary material for: Rab11 modulates α-synuclein-mediated defects in synaptic transmission and behaviour
Source: Hum Mol Genet. 2014 Oct 9;24(4):1077–91. doi: 10.1093/hmg/ddu521 (PMC4986550; doi:10.1093/hmg/ddu521)
Supplement: Supplementary Data [file supp_ddu521_ddu521supp.pdf]

Supplementary Data

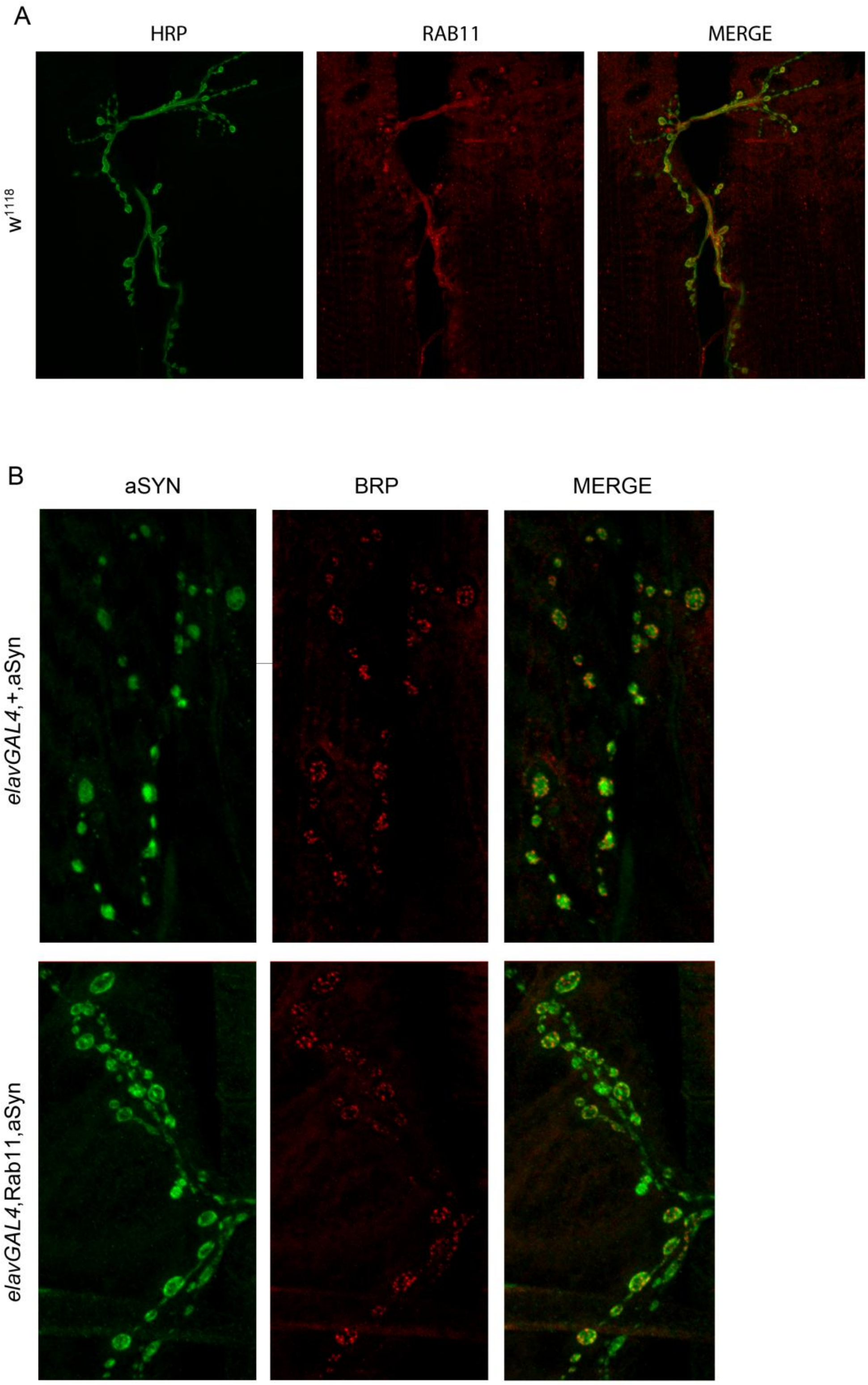

Figure S1. Endogenous Rab11 localises to the larval NMJ. Immunostaining in third instar larvae detects Rab11 expression at NMJs (A). The distribution of *bruchpilot* (BRP) is not affected in larval NMJs expressing aSyn or Rab11 + aSyn in Model 1 flies (B).

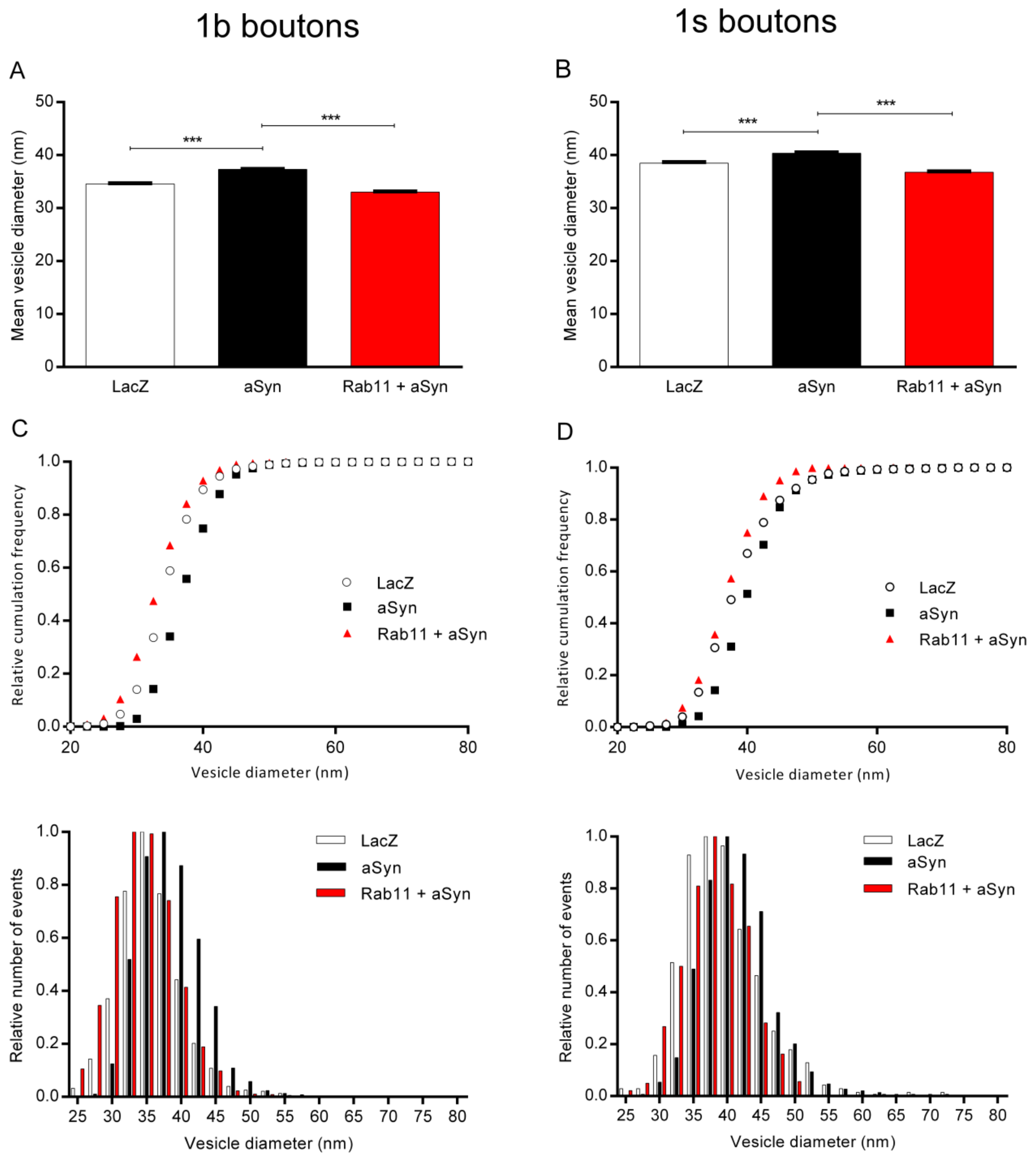

Figure S2. aSyn transgenic flies exhibit enlargement of synaptic vesicles. Pan-neuronal expression of aSyn in Model 2 flies increases vesicle diameter in both 1b and 1s boutons compared to LacZ-expressing controls (A and B, respectively). Relative cumulative frequency (upper graph) and histogram (lower graph) plots of synaptic vesicle size for 1b (C) and 1s (D) boutons indicate a right shift in aSyn larvae compared with LacZ control larvae. The overexpression of Rab11 restores the vesicle size to control level. Data are Mean ± SEM. ANOVA with Newman-Keuls *post hoc* tests. *** P < 0.001.

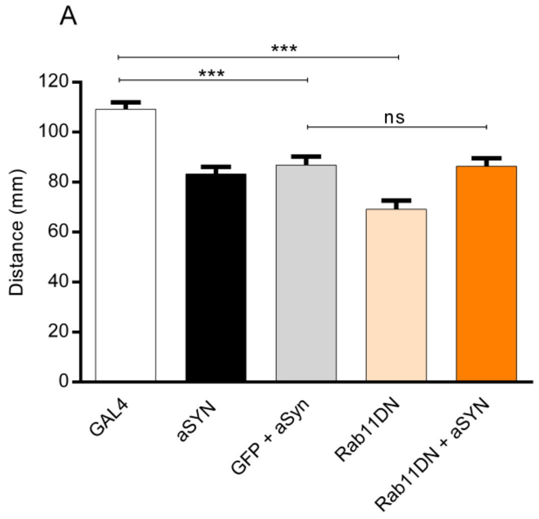

Figure S3. Rab11S25N overexpression in aSyn larvae does not rescue crawling behaviour. Mean distance travelled by Model 2 larvae expressing aSyn, GFP + aSyn, Rab11S25N and Rab11S25N + aSyn in the motorneurons (A). While Rab11S25N decreases the travelled distance in WT larvae, its expression does not influence aSyn larvae. Data are Mean ± SEM. ANOVA with Newman-Keuls *post hoc* tests. *** $P < 0.001$. $N = 30$ for each genotype.

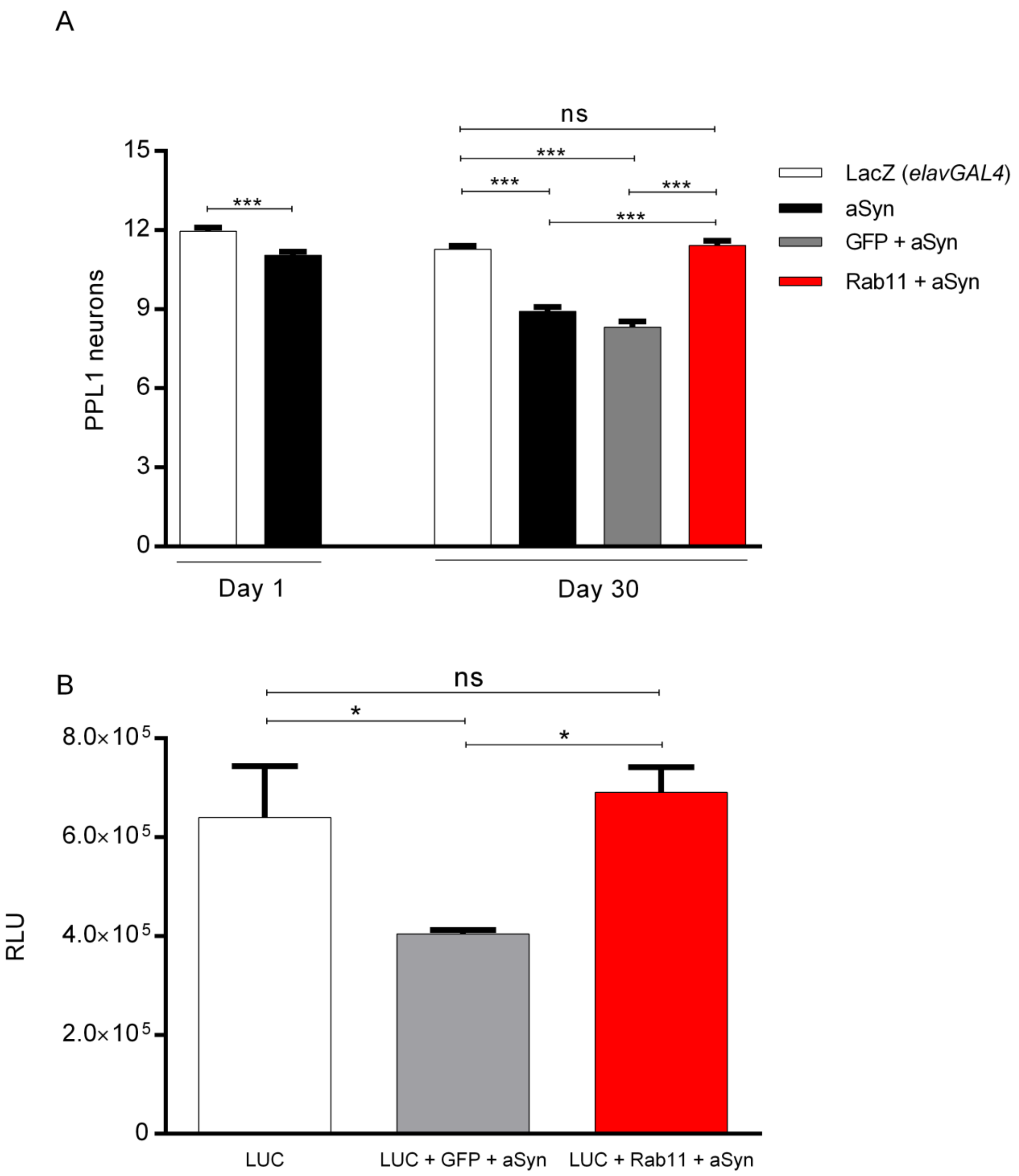

Figure S4. Dopaminergic neuron loss in aSyn transgenic flies is rescued by Rab11 overexpression. Expression of aSyn in Model 2 flies by *elavGAL4* leads to TH-positive cell death at day 1 and day 30 compared to flies expressing LacZ (A). Neurodegeneration is restored to control levels by the overexpression of Rab11 (N = 16-68 hemispheres). Luciferase intensity measurements in 30 day old Model 2 transgenic flies expressing aSyn pan-neuronally (B). GFP + aSyn flies display a reduction of luciferase signal which is rescued by the overexpression of Rab11 (N = 4 samples). Data are Mean ± SEM. ANOVA with Newman-Keuls *post hoc* tests. * $P < 0.05$, *** $P < 0.001$; ns = not significant.

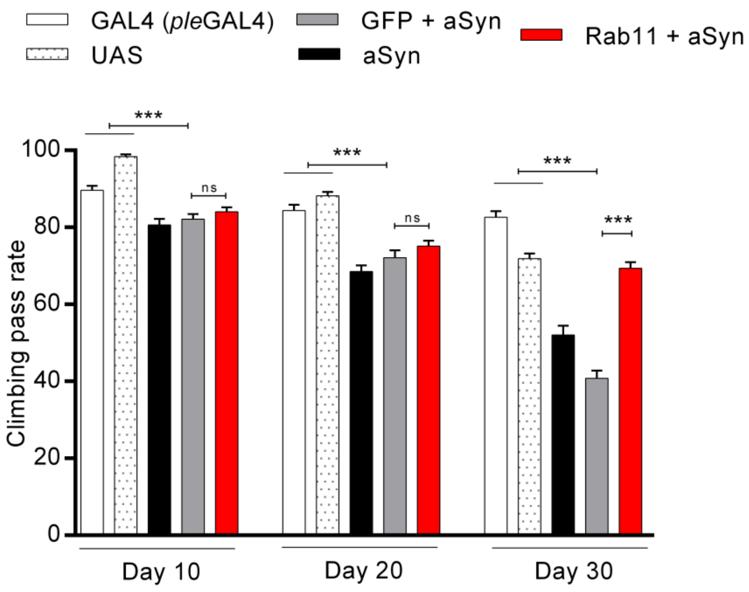

Figure S5. aSyn-dependent adult fly phenotypes are ameliorated by Rab11 overexpression. Mean climbing pass rate for flies expressing aSyn*,* GFP + aSyn + GFP and Rab11 + aSyn in the dopaminergic neurons via the *pleGAL4* driver (N = 50 – 60 per condition). aSyn expression causes a reduction in climbing at all post-eclosion ages which are strongly rescued by Rab11 overexpression. Data are Mean ± SEM. ANOVA with Newman-Keuls *post hoc* tests. *** P < 0.001 and ns = not significant.

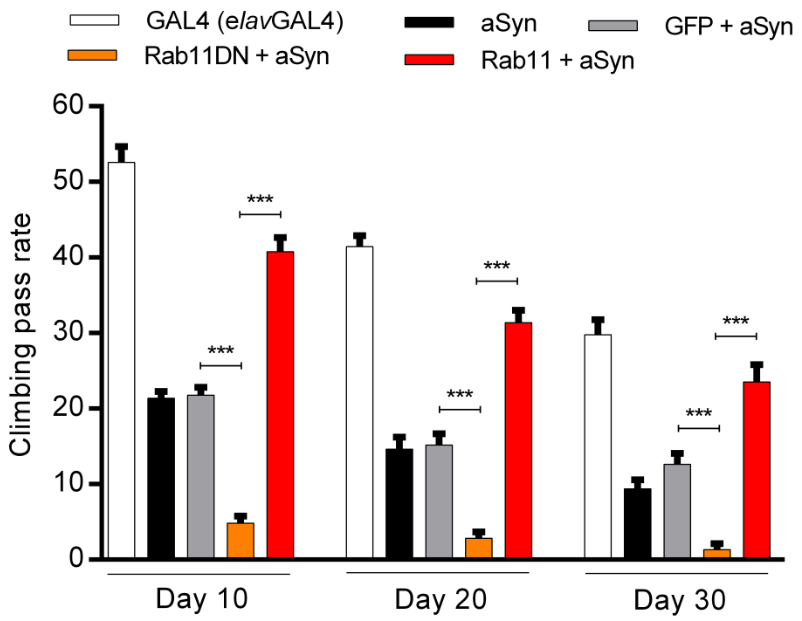

Figure S6. aSyn climbing behaviour is worsened by Rab11S25N overexpression. Mean climbing pass rate for flies expressing pan-neuronally aSyn*,* GFP + aSyn + GFP and Rab11 + aSyn by *elav*GAL4 driver (N = 50 – 60 per condition). Rab11S25N overexpression causes a reduction in climbing ability in aSyn flies at all post-eclosion ages. Data are Mean ± SEM. ANOVA with Newman-Keuls *post hoc* tests. *** P < 0.001 and ns = not significant.
